# Examining harmful impacts of the COVID‐19 pandemic and school closures on parents and carers in the United Kingdom: A rapid review

**DOI:** 10.1002/jcv2.12095

**Published:** 2022-08-19

**Authors:** Hope Christie, Lucy V. Hiscox, Sarah L. Halligan, Cathy Creswell

**Affiliations:** ^1^ Department of Clinical Psychology University of Edinburgh Edinburgh UK; ^2^ Department of Psychology University of Bath Bath UK; ^3^ Department of Experimental Psychology & Department of Psychiatry Oxford University Oxford UK

**Keywords:** caregivers, COVID‐19, families, mental health, parents, school closures, well‐being

## Abstract

**Background:**

As a result of the COVID‐19 pandemic, school closures meant that for many households, home and school environments became intertwined. Parents and carers found themselves taking on the role as de‐facto educators, as well as balancing working from home and caring for additional members of the household. Understanding the full extent of the effects incurred by parents and carers during school closures is vital to identifying and supporting vulnerable families. This rapid review aimed to appraise the available evidence on the potential effects of the COVID‐19 pandemic on UK parents and carers.

**Methods:**

Searches for academic literature were conducted using Proquest Central, Scopus, and Google Scholar between 21st and 28th April 2021 using search terms describing “parents and carers”, “COVID‐19” and the “UK”. Additional literature was identified on relevant parents and carers' organisations websites including charity reports.

**Results:**

Thirty‐two articles were found relating to harms affecting parents and carers in the UK High levels of psychological distress, including anxiety and depression, were consistently identified in the general parent population, and especially in parents caring for a child with special educational needs and/or neurodevelopmental disorders (SEN/ND). Charity reports indicated that many parents, especially those from an ethnic minority background and kinship carers, were worse off financially and with food insecurities, whereas empirical evidence showed that mothers were more likely to initiate furlough for themselves compared with fathers or childless women. Domestic abuse support services also reported a sharp rise in demand during lockdown restrictions, and practitioners reported an increase in child and adolescent violence towards parents.

**Conclusions:**

Given the known impacts of parental stress, mental health problems, domestic violence and financial hardship on children's development, it is critical that these findings are taken into account in case of future pandemics to minimise harms both to parents and their families.

## Graphical abstract

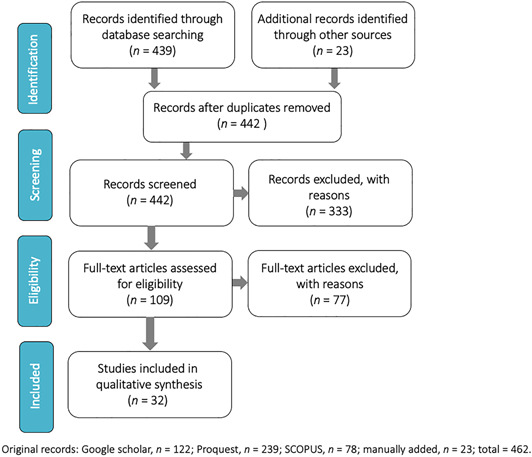

As a result of the COVID‐19 Pandemic families were encouraged to stay at home as the result of school closures and disruption to daily routine. The current rapid review investigates the impact of these disruptions on parents and caregivers across the United Kingdom. Results found a number of impacts including parent mental health, domestic violence and financial hardship, all of which can have substantial effects on children's development.

**Key points**

- The COVID‐19 pandemic has caused global disruption, as well as causing illness, long standing health complications and even death. Across the United Kingdom the country went into a nationwide lockdown, which included school closures and stay at home initiatives.
- This rapid review investigated the potential impacts of the school closures and national lockdowns on parents and carers in the UK.
- Findings were taken from empirical and emerging evidence and highlighted high levels of parental psychological distress as well as some groups being more vulnerable than others (e.g., single parents and those with a child with Special Educational and/or Neurodevelopmental Disorders).
- Findings also emphasised some parent and carer groups (e.g., ethnic minorities and kinship carers) were worse off financially and experienced food insecurity.
- Potential interventions and/or support are required to support parents and minimise harms to both parents and their families.

## INTRODUCTION

The Coronavirus pandemic (COVID‐19) has been recognized as a globally disruptive health crisis that has thus far resulted in >497 million confirmed cases, and over 6 million deaths worldwide mandatory lockdowns, movement restrictions, and enforced physical distancing measures; and individuals have dealt with stress, fear and uncertainty of virus spread and severity. The full ramifications of the pandemic are yet to be fully seen; however, research predicts long‐standing effects at an individual and wider societal level.

The lives of millions of parents and their children have been affected not only by the health and economic implications of COVID‐19 pandemic, but also by school closures. For most households, home and school environments became intertwined, with most schools unprepared to support home‐schooling on such a mass scale. In the UK, schools closed to in person learning on 20 March 2020, except for schools for children of key workers and children with special needs, with phased reopening for limited year groups beginning on 1 June 2020. Across England, Wales, and Northern Ireland, 94% of schools had no more than 20% of their pupils attend in‐person teaching during the first national lockdown (National Association of Head Teachers, 2020). Consequently, for many families, the teaching and learning of their children became the responsibility of parents and carers, which in tandem with working from home, presented numerous challenges. Understanding the full extent of harmful effects for parents during lockdown is vital to identify families who may have been particularly affected, to understand how best to support parents now lockdown restrictions across the UK have eased, and to inform practice in case of future events that may cause widespread disruption to children's access to school. The aim of this rapid review was to provide a comprehensive overview of potential harmful effects of the COVID‐19 pandemic on UK parents.

## METHODS

To ensure the rapidity of this review, the focus of searches was directed by a pre‐determined ‘harms’ list provided by the UK Government's Scientific Advisory Group for Emergencies (SAGE) and the Department for Education (see Appendix S1 in the Supporting Information).

### Search strategy

‘Harms’ were defined as psychological, social, emotional or contextual impacts of the pandemic, lockdown restrictions and school closures that may have affected parents and their families. Searches for academic literature were conducted between the 21st and 28th April 2021 and included terms related to “harms” and “COVID‐19” (see Table 1). Hand searches for grey literature were performed on Google and on relevant parent and carer organisation websites using the same search terms wherever possible. Search sources were limited to Proquest Central, Scopus, and Google Scholar using free text and subject headings search terms describing parents, COVID‐19 and the UK. The IPPO Living Map was also searched which was updated monthly with newly published systematic reviews relating to the COVID‐19 pandemic. Inclusion and exclusion criteria are listed below (Table 2).

**TABLE 1:** Search string used for literature searching

(("parent s"[All Fields] OR "parentally"[All Fields] OR "parentals"[All Fields] OR
"parented"[All Fields] OR "parenting"[MeSH Terms] OR "parenting"[All Fields] OR
"parents"[MeSH Terms] OR "parents"[All Fields] OR "parent"[All Fields] OR
"parental"[All Fields] OR ("mother s"[All Fields] OR "mothered"[All Fields] OR
"mothers"[MeSH Terms] OR "mothers"[All Fields] OR "mother"[All Fields] OR
"mothering"[All Fields]) OR ("father s"[All Fields] OR "fathered"[All Fields] OR
"fathers"[MeSH Terms] OR "fathers"[All Fields] OR "father"[All Fields] OR
"fathering"[All Fields]) OR ("caregiver s"[All Fields] OR "caregivers"[MeSH Terms]
OR "caregivers"[All Fields] OR "caregiver"[All Fields] OR "caregiving" OR
“Guardian” [all Fields]))
**AND**
("sars cov 2"[MeSH Terms] OR "sars cov 2"[All Fields] OR "covid"[All Fields] OR
"covid 19"[MeSH Terms] OR "covid 19" OR “lockdown” [All Fields]))

*Note*: Search terms were left purposefully broad in order to capture a significant proportion of relevant literature.

**TABLE 2:** Summary of inclusion and exclusion criteria

Inclusion	Exclusion
Published since November 2019	Study focus was not on parents and carers
Related to COVID‐19	Data were collected from participants residing outside the United Kingdom
Reported data UK or/and Northern Ireland populations	Age of children (if reported) were not school age (i.e., 18 years and under)
Studies involving parents or carers and their children as the key study population	Data was not collected or was covering the COVID‐19 pandemic
Reported on one (or multiple) harms found in the DfE list	

### Study eligibility criteria

**FIGURE 1:**
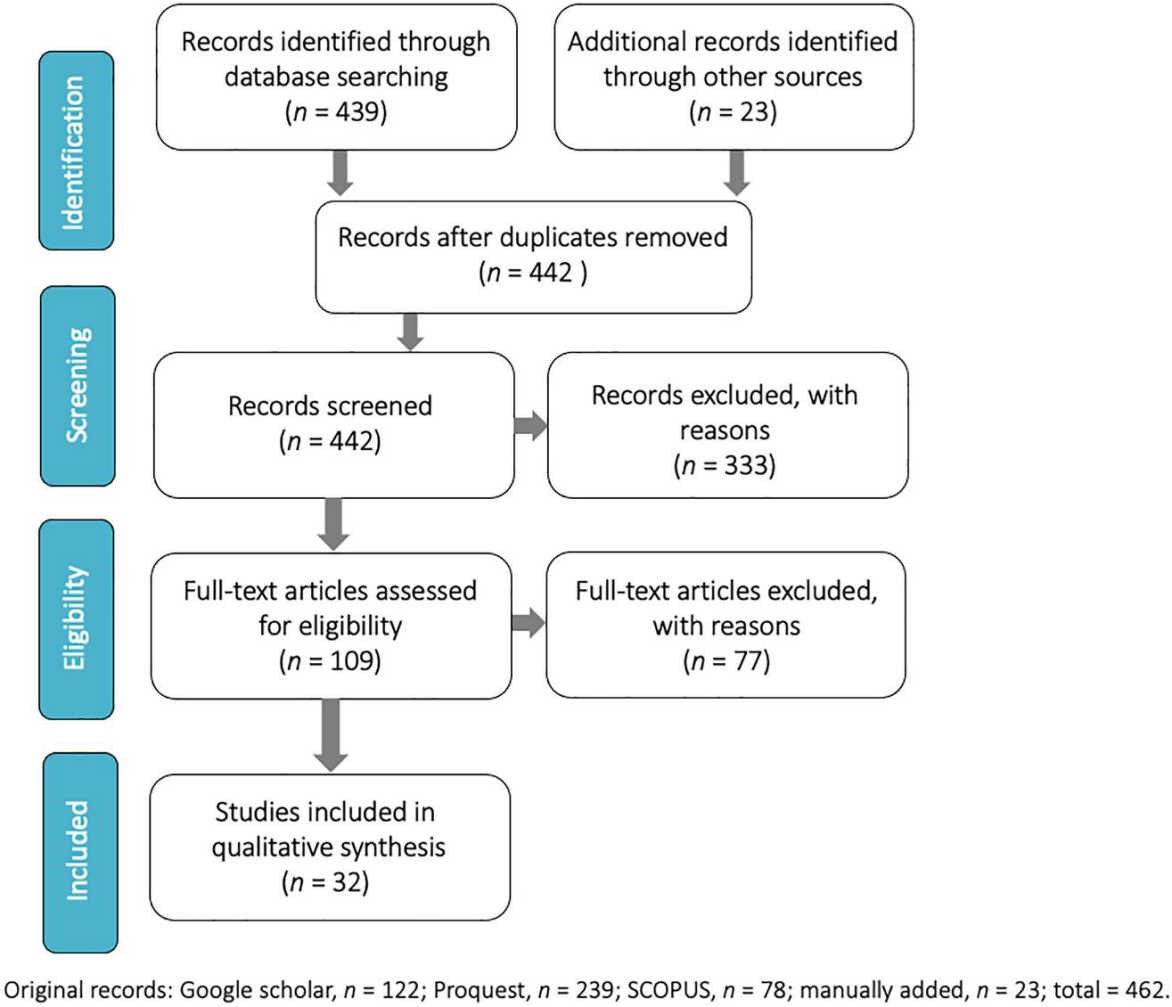
PRISMA flow diagram showing manuscript selection

We captured studies of UK parents/carers of children 18 years and younger relating to potential harms caused by the COVID‐19 pandemic. The study selection process is summarized in the PRISMA diagram (Figure 1), with reasons for exclusion.

### Study selection, data extraction, risk of bias assessment

Authors HC and LH each screened half of the articles, checking 10% of the other's screening decisions to ensure consistency. Any disagreements were discussed and resolved by SH and CC. In addition, SH and CC contributed to results summaries, ensuring information was presented in a clear, concise, and unbiased format. [Correction added on 22 August 2022, after first online publication: Authors' names have been included in this version.]

## RESULTS

In total, 32 papers (23 empirical; nine emerging evidence) were included in the final review. Three major themes were identified: changes to parental mental‐health and well‐being; earning capacity changes; and exposure to physical harms in the domestic setting. Further details of each paper and the main outcome of the quality assurance are provided in Table S1 and S2.

### Mental health and well‐being

Twenty‐five journal articles and charity reports examined the mental health and well‐being of UK parents.

#### Psychological distress in general parent populations

Six articles documented increases in psychological distress in UK parents in relation to lockdown restrictions. Two used data from the UK Household Longitudinal Study (UKHLS; Pierce et al., 2020; Xue & McMunn, 2021), one used data from the Born in Bradford study (Dickerson et al., 2021) and three from the Co‐Space study (COVID‐19: Supporting Parents, Adolescents and Children during Epidemics; McElroy et al., 2020; Shum et al., 2020; Waite et al., 2020).

Data from UKHLS collected during the first national lockdown found that levels of mental distress were found to have particularly increased in parents of pre‐school children when compared to levels of mental distress in 2018/29 (Pierce et al., 2020). More specifically, a 1.45‐point increase on scores from the General Health Questionnaire (maximum score 36) compared to only a 0.33 increase among adults without children. Furthermore, mothers were more likely than fathers to have reduced their working hours due to increased time spent on childcare, and mothers who spent long hours on both housework and childcare were more likely to report increased levels of psychological distress (Xue & McMunn, 2021). It was also found that lone mothers reported particularly high levels of distress (Xue & McMunn, 2021).

The Born in Bradford study comprised 2144 parents (95% mothers) from a prospective birth cohort study who responded to a questionnaire survey administered during April‐June 2020 when the UK was in its first national lockdown (Dickerson et al., 2021). Many parents (74%) had children who were aged between 9 and 13 years. Clinically significant symptoms of depression were reported by 19% (*n* = 843) of respondents, and 16% of respondents reported clinically significant symptoms of anxiety. Moderate and severe parental depression and anxiety were associated with financial insecurity, as well as unemployment, poor quality housing, having self‐isolated at some point, poor health, and a lack of social support. Nearly half of the parent sample (47%) also reported a decrease in physical activity compared to pre‐lockdown levels, with lower levels of exercise being associated with poorer mental health outcomes.

A report from the longitudinal Co‐SPACE study documented the mental health of a convenience sample of 6246 parents who completed monthly measures between April 2020 and March 2021 (Shum et al., 2020, 2021; Waite et al., 2020), during periods of lockdown and increased restrictions, relative to periods when restrictions were relaxed, with the highest levels of distress being documented between November 2020 and February 2021, a period of regional restrictions followed by a second national lockdown (Shum et al., 2021). Single adult households and low‐income families had elevated mental health symptoms throughout the whole period assessed. Parents children aged 10 years or younger reported particularly high levels of stress when restrictions were highest, whereas parents with older children reported more depressive symptoms, especially during the summer. Parents of older children were more likely to be stressed about their children's education and future than those of young children. Finally, McElroy et al., 2020 reported that, during the first 6 weeks of the first lockdown, pandemic‐related anxieties among parents were multi‐faceted, relating to both disease anxiety (e.g., catching, transmitting the virus) and consequence anxiety (e.g., impact on economic prospects).

#### General parental wellbeing

Two reports documented general parental well‐being during the pandemic (El‐Osta et al., 2021; Royal Foundation of the Duke and Duchess of Cambridge, 2020).

As a part of early years research commissioned by the Royal Foundation of the Duke and Duchess of Cambridge (2020), a convenience online survey of 1000 parents of 0–5‐year‐olds was carried out in October 2020. Compared data collected between September 2019 and February 2020, the number of parents that reported feeling lonely dramatically increased; from 38% prior to the pandemic to 63% by October 2020. Parents also reported needing support for a wide range of issues including child health, nutrition, behaviour, and sleep, which constituted a large source of their stress. Almost two in five parents (37%) expected that the pandemic would have a negative impact on their long‐term mental health.

In the second cross‐sectional study, approximately half of the participating 1214 parents of school‐age children in the UK felt they lacked companionship, had feelings of being left out, felt isolated, and lonely during the first 100 days of lockdown (El‐Osta et al., 2021). Being female, parenting a child with special needs, a lack of dedicated space for distance learning, unemployment, disruption of sleep patterns and low levels of physical activity were each associated with higher levels of loneliness.

#### Impacts of home‐schooling

Seven reports identified home‐schooling requirements as a factor in parent's wellbeing. In a sample of 4234 UK parents, 28% agreed that home‐schooling negatively affected their wellbeing in April 2020 and 36% reported detrimental effects on relationships, with these figures rising to over 50% respectively by January 2021 (Office for National Statistics, 2021).

In Northern Ireland (NI), approximately half of 1905 parents homeschooling post‐primary school pupils reported difficulties in managing both their mental and physical well‐being (Bones et al., 2020). An additional survey of parents homeschooling in NI, almost 80% of 2002 parents reported a negative impact on their own mental health and wellbeing, with the most acute impact reported by parents who were also working from home (Purdy & Harris, 2021). Many parents also expressed fear and anxiety due to their child's disrupted education. Moreover, across the U.K. (508 families surveyed), 57% of parents of children with mental health conditions or additional needs (which was collected via parent report and included dyslexia, depression, anxiety, and autism, for a full list see Thorell et al., 2021) reported that homeschooling had a negative impact on themselves, compared with 37% of parents who have children without a mental health condition (Thorell et al., 2021).

#### Parents of children with pre‐existing medical conditions

171 parents and caregivers of children with cancer were surveyed during the early stages of the pandemic, when children with cancer were designated as ‘clinically extremely vulnerable’. While no comparative data were available, Darlington et al. (2020) reported that 85% of participating parents were worried about the virus and 90% were concerned about transmitting the virus to their child. For two‐thirds of respondents, hospital was no longer considered a safe place, and parents were worried about suboptimal cancer care. Parents, and particularly single parents, also described difficulties coping with the uncertainty of the situation, lack of control, and limited support being in place.

#### Parents of children with special educational needs and/or neurodevelopmental disorders (SEN/ND)

Findings from several separate surveys of parents with SEN/ND children, also show how parents felt overwhelmed when trying to home‐school their children, highlighting that they felt unprepared, inadequate, and worried that they were letting their child down (Greenway & Eaton‐Thomas, 2020; Toseeb et al., 2020). Further, four surveys indicated that parents of children with SEN/ND may have experienced particular detriments to their wellbeing during lockdown (Gillespie‐Smith et al., 2021; Shum et al., 2021; Thorell et al., 2021; Waite et al., 2020).

In survey data collected from the first 5000 parent/carers who participated in the Co‐SPACE study, the majority (51.5%) of the 871 parents with children with SEN/ND felt stressed about their child's behaviour compared to only 4% of parents without a SEN/ND child (Waite et al., 2020). Parents of children with SEN/ND also reported that they would benefit from additional support with a larger proportion (relative to parents without SEN/ND) stating they needed support managing their child's emotions, behaviour, education, and family relationships. Parents of SEN/ND children reported elevated anxiety, depression, and stress symptoms throughout the period from March 2020 to January 2021 that spiked when restrictions were strictest. Over time, more parents of children with versus without SEN/ND were stressed about their child's behaviour (48% vs. 24%), wellbeing (63% vs. 39%), screen time (45% vs. 37%), education (54% vs. 34%), and future (52% vs. 32%; Shum et al., 2021).

In a separate survey of 508 UK families (of which 37.4%, *n* = 189 had children with SEN), parents of children with SEN reported feeling stressed (69.5%), socially isolated (69.2%), and experiencing conflict with their child (40.4%) during lockdown, and each of these problems was more common than in families with typically developing (TD) children (Thorell et al., 2021). A study of parents recruited during April‐May 2020 (*n* = 43 with ND; *n* = 67 with TD) showed that challenging behaviours were associated with psychological distress in parents of children with ND during the COVID‐19 pandemic (Gillespie‐Smith; under review).

#### Studies of women of infants

Two studies have examined maternal postnatal mental health during the pandemic (Fallon et al., 2021; Vazquez‐Vazquez et al., 2021).

In a cross‐sectional study with a convenience sample of 614 mothers (with infants aged between birth and 12 weeks) conducted between April and May 2020, Fallon et al., 2021 reported that 43% of participating mothers scored above the clinical cut off for depression, and 61% exceeded the clinical cut‐off for anxiety, although data were not available prior to the pandemic. Further, results from 1365 women who delivered before (May 2020) or during (June 2020) lockdown suggest that hospital facilities were continuing to implement measures during lockdown such as promoting early mother‐baby contact and initiation of breastfeeding, thereby minimizing psychological stress and detrimental effects on feeding and bonding (Vazquez‐Vazquez et al., 2021). Between groups, there was also no significant difference in the support mothers received by a mental health professional, and in fact women who delivered during lockdown reported greater contact with a health professional and Mother & Baby or breastfeeding groups. Of concern, 57% of women who delivered before lockdown experienced a decrease in infant feeding support during the subsequent lockdown, which should be noted given that there is evidence that the quality or lack of breastfeeding support is related to an increased risk of later postnatal depression (Chaput et al., 2016).

### Earning capacity changes

Seven reports documented the extent to which UK parents were affected by the economic repercussions of COVID‐19.

#### Economic harms

According to a poll by the *Save the Children* charity conducted in the second week of lockdown, 10% of 1002 participating parents of 6–18 year olds had to leave their jobs completely, while 29% were forced to reduce working hours or take unpaid leave due to increased childcare needs (Save the Children, 2020). In survey data collected from 8940 respondents in May 2020, among parents with at least one child living in the house (*n* not specified) a larger proportion of mothers were likely to initiate furlough when compared to childless women, and a greater proportion of mothers initiated furlough compared to fathers (Adams‐Prassl et al., 2020). In contrast, there were no gender differences in who initiated furloughing among respondents without children. In another survey of 2144 parents (95% mothers; 5% partners) that compared pre‐to‐post lockdown levels of financial insecurity, 33% of the sample stated their financial status was worse than it was 3 months previously (from Feb to May 2020). This varied by participants' ethnicity; 37% of those with a Pakistani heritage disclosed they were worse off financially compared to 26% of White British participants (Dickerson et al., 2021).

### Physical harms—Violence in the home

Lockdown conditions appeared to substantially exacerbate domestic abuse. Information regarding physical harms came from reports from charities and local authorities (Condry et al., 2020; Newbury et al., 2020). A lack of empirical evidence covering this topic means the level of information reported is limited. However, charities and local authorities reported an increase in calls and visits to charity helplines and websites regarding domestic violence support (in May 2020 Refuge's National Domestic Abuse Helpline report visits to their website increased by 950% above pre‐pandemic levels); yet a decrease in attendances to accident and emergency departments. Across police forces in Wales, incidences of reporting of domestic abuse was also reported to have reduced by 15%, compared to the previous year (Newbury et al., 2020). Another report did note that, of 104 parents surveyed, 70% reported an increase in violent behaviour by their child towards them (Condry et al., 2020).

## DISCUSSION

We identified harms to parents and carers in three main areas: mental health and wellbeing; earning capacity changes; and physical harms in the home due to both domestic violence and child and adolescent violence towards parents.

Overall, we found that UK parents and carers self‐reported elevated levels of psychological distress, including anxiety, and depression during lockdown restrictions, coinciding with the timing of home‐schooling. Evidence also suggests that being female, possessing a lower income, being a single parent, having a child with SEN/ND, and/or being from an ethnic minority background may have further exacerbated parental psychological distress, highlighting groups that may need additional support to support recovery following the pandemic and incase of similar future events. However, studies were typically cross‐sectional surveys with non‐representative samples and without pre‐pandemic comparison groups, making it difficult to fully ascertain the extent to which the pandemic has had a negative impact on parent's mental health. In one key longitudinal study with a nationally representative sample and data obtained both before and during lockdown, significantly higher rates of mental distress were particularly reported by parents with young children relative to those without children, providing the clearest indication yet that parents are likely to have been disproportionally affected by school lockdown measures (Pierce et al., 2020). In addition, parental loneliness and isolation was also highlighted as a potential harm. This is a cause for concern given that loneliness has been shown to have a longer‐term impact on mental and physical health (Masi et al., 2011). Together, these findings highlight considerable cause for concern for the consequences to children, as there is strong evidence that links parental mental health and well‐being as a critical factor in effective parenting (Cuijpers et al., 2015) and children's cognitive and social development (Masi et al., 2011). As a result, parents, and particularly those identified as most at‐risk, should be prioritized for support to be in the best position to support their children.

We found robust evidence to suggest that the pandemic has had a substantial negative effect on the earning capacity of UK parents, and particularly mothers (Adams‐Prassl et al., 2020). Future work will need to assess whether loss of earnings to parents during school closures will have a long‐term harmful effect by pushing more families into poverty and lowering socioeconomic status (Dickerson et al., 2021), which has been linked to significant reductions in life expectancy (Stringhini et al., 2017). In addition, families' financial situation or SES has been shown to cause educational losses for their children (Bayrakdar & Guveli, 2020; Engzell et al., 2021) and further longitudinal studies are needed to examine how this initial loss of learning may translate to long‐term attainments and future prospects.

For some families, increased time in the family home may have also given rise to an increased risk of domestic violence. While formal reporting of domestic violence and hospital admissions decreased during the first national lockdown, charities have reported substantial increases in calls relating to domestic abuse (Newbury et al., 2020). For children in the home, witnessing domestic violence can negatively affect their psychological, emotional, and social development, increasing disruptive behaviours, which may in turn cause difficulties for them at school (Carrell & Hoekstra, 2010). According to self‐reports and support services, there has also been an increase in the prevalence of child/adolescent to parent violence (although data from police reports were not conclusive). Several charity reports describe increased aggressive, violent, or challenging behaviours from children towards their parents (Condry et al., 2020). While this has been speculated to be related to experiences of confinement and coerced proximity, changes in structure and routine, fear and anxiety, and lack of access to support, future studies will need to determine the root causes of such violence by children towards their parents. Regardless, the presence of these externalizing behaviours in children indicates urgent need for support for families who remain affected (Carrell et al., 2018).

Across all harms reported, we have seen an exacerbation of pre‐existing gender inequalities such that mothers appear to have been hardest hit during the lockdown compared to fathers. Mothers were more likely than fathers to report concerns that the pandemic would have a negative impact on their long‐term mental health, and they spent more time on childcare responsibilities than fathers which led to both an increase in psychological distress and a loss of earnings (Darlington et al., 2020; Office of National Statistics, 2021). We also found that black and minority ethnic women were disproportionately affected by a loss of earnings due to cuts to public services (Ferrer & Parvez, 2020).

Strengths of this review include that literature searches were extensive, included most relevant data sources, and were conducted by research specialists. Data was also handled in a systematic and critical way with evidence reviewed and synthesized by topic experts. Several of the studies identified drew upon large longitudinal data sets that were able to report changes directly from pre‐to‐post pandemic and which were directly relevant to harms experienced by the parent population. As for limitations, the short‐time frame restricted sources of additional literature such as harms experienced to parents from outside of the UK The identification of studies included was also not undertaken fully independently. Not all the available evidence was peer‐reviewed, and a large body of the articles were based on cross‐sectional surveys that did not always have an appropriate comparison group and/or were unable to accurately state the specific impact of the pandemic due to capturing only one moment in time. It may be too early to fully see the extent of harms to parents and we anticipate that more, potentially unanticipated harms may come to light as new data emerge. Finally, our focus was specifically on harms however it is important to acknowledge that some studies have also identified benefits, for example, spending more time together as a family (Royal Foundation of the Duke and Duchess of Cambridge, 2020).

## CONCLUSIONS

This review has found evidence of harms to UK parents due to national lockdown restrictions (which coincided with school closures) from 32 research articles, reports, and charity surveys. Identified harms fell into three main categories: mental‐health and well‐being; earning capacity changes; exposure to physical harms in the domestic setting. Overall parents appear to have suffered disproportionately compared to non‐parents. Notably, for many of these outcomes there is evidence that parental gender and social group imbalances may have widened, with mothers, those with lower SES, and black and ethnic minority groups being affected to a greater extent. Research evidence published prior to the pandemic suggests that parental harms are likely to have negative consequences for children and the challenge of living in a pandemic may have exacerbated these consequences for children. Further research is needed to understand the impacts on children's development, wellbeing, and academic attainment. Further, interventions implemented to address or reduce the harms reported by parents will need to be studied carefully to ensure they are successful in supporting parents' and their children's well‐being and long‐term development.

## AUTHOR CONTRIBUTIONS

Hope Christie and Lucy V. Hiscox carried out the following tasks: searching of articles; screening of all search results; data extraction and synthesis of findings; producing the main and technical reports. Cathy Creswell and Sarah L. Halligan provided support and guidance throughout the process, edited drafts and provided input into the final document.

## CONFLICTS OF INTEREST

The authors have declared that they have no competing or potential conflicts of interest.

## ETHICAL CONSIDERATIONS

No ethical approval was required for this review.

## Supporting information

Supplementary Material

## Data Availability

Data sharing is not applicable to this article as no new data were created or analyzed in this study.
