# Supplementary material for: Examining harmful impacts of the COVID‐19 pandemic and school closures on parents and carers in the United Kingdom: A rapid review
Source: JCPP Adv. 2022 Aug 19;2(3):e12095. doi: 10.1002/jcv2.12095 (PMC9537939; doi:10.1002/jcv2.12095)
Supplement: Supplementary file 1 — Supplementary Material [file JCV2-2-e12095-s001.docx]

**Supporting Information**

**Appendix S1.** Pre-determined ‘harms’ list provided by UK Government’s Scientific Advisory Group for Emergencies (SAGE) and the Department for Education

The DfE provided the following lists of harms to consider both in the short- and longer-term:

- Mental Health
- Well-Being & Development
- Physical Health
- Nutrition
- Misuse of Substances
- Domestic Violence
- Support Service Access
- Indirect Groups at Risk (e.g., those with extended caring responsibilities)
- Vulnerable children and SEND children
- Learning loss / Educational Knock-on Effect
- Immediate Earning Capacity Changes

In addition, these longer-term harms were also considered.

- Gender & Social Group Imbalance Widening
- Changes in socioeconomic status (SES)

**Table S1.** Overview of evidence of harms with QA (32 studies)

Presented in order of main text

| Count* | Sub-category | Evidence source (reference) | Study type | Strength of the evidence |
| --- | --- | --- | --- | --- |
| 1. Mental health and well-being | | | | |
| 27 | Psychological distress | Pierce, M et al., 2020. Mental health before and during the COVID-19 pandemic: a longitudinal probability sample survey of the UK population. | Secondary analysis study (data from Understanding Society Covid-19 survey) | Medium – some limitations |
|  |  | Xue, B. and McMunn, A., 2021. Gender differences in unpaid care work and psychological distress in the UK COVID-19 lockdown. | Secondary analysis study (data from Understanding Society Covid-19 survey) | Medium – some limitations |
|  |  | Dickerson, J. et al., 2021. Experiences of lockdown during the Covid-19 pandemic: descriptive findings from a survey of families in the Born in Bradford study. | Cross-sectional (Online survey) | Medium – some limitations |
|  |  | Waite, P. et al., 2020. Report 02: COVID-19 worries, parent/carer stress and support needs, by child special educational needs and parent/carer work status. | Cross-sectional (Online survey) | Medium – some limitations |
|  |  | Shum, A. et al., 2020. Report 07: Changes in parents’ mental health symptoms and stressors from April to December 2020. | Longitudinal  (Online survey) | Strong |
|  |  | McElroy, E. et al., 2020. Demographic and health factors associated with pandemic anxiety in the context of COVID-19. | Cross-sectional (Online survey) | Medium – some limitations |
|  | Well-being | Royal Foundation of the Duke and Duchess of Cambridge, 2020. State of the nation: understanding public attitudes to the early years. IPSOS MORI. | Longitudinal (Online survey) | Strong |
|  |  | El-Osta, A. et al., 2021. How is the COVID-19 lockdown impacting the mental health of parents of school-age children in the UK? | Cross-sectional (Online survey) | Strong |
|  | Impacts of home-schooling | Office for National Statistics, 2021. *Coronavirus and the social impacts on Great Britain: 19 February 2021* [Online]. | Survey | Strong |
|  |  | Bones, O.C. et al., 2020. *Ulster University Northern Ireland Parent Surveys: Experiences of Supporting Children's Home Learning during COVID-19.* | Cross-sectional (Online survey) | Medium – some limitations |
|  |  | Purdy, N.et al., 2021. *Northern Ireland Survey of Parents/Carers on Home-Schooling during the COVID-19 crisis 2021.* | Cross-sectional (Online survey) | Medium – some limitations |
|  |  | Asbury, K. et al., 2020. How is COVID-19 Affecting the Mental Health of Children with Special Educational Needs and Disabilities and Their Families? | Cross-sectional (Online survey) | Medium – some limitations |
|  |  | Greenway, C.W. and Eaton-Thomas, K., 2020. Parent experiences of home-schooling children with special educational needs or disabilities during the coronavirus pandemic. | Cross-sectional (Online survey) | Medium – some limitations |
|  |  | Toseeb, U.A., K; Code, A; Fox, L; Deniz, E, 2020. Supporting Families with Children with Special Educational Needs and Disabilities During COVID-19. | Cross-sectional (Online survey) | Medium – some limitations |
|  |  | Thorell, L.B. et al., 2021. Parental experiences of homeschooling during the COVID-19 pandemic: differences between seven European countries and between children with and without mental health conditions. | Cross-sectional (Online survey) | Medium – some limitations |
|  | Parents of children with pre-existing medical conditions | Darlington, A.-S. et al., 2020. COVID-19 and children with cancer: Parents’ experiences, anxieties, and support needs. | Cross-sectional (Online survey) | Medium – some limitations |
|  | Parents of children with SEN/ND | Waite, P. et al., 2020. Report 02: COVID-19 worries, parent/carer stress and support needs, by child special educational needs and parent/carer work status. | Cross-sectional (Online survey) | Medium – some limitations |
|  |  | Shum, A. et al., 2021. Report 09: Update on children’s & parents/carers’ mental health; Changes in parents/carers’ ability to balance childcare and work: March 2020 to February 2020. | Longitudinal (Online survey) | Strong |
|  |  | Thorell, L.B. et al., 2021. Parental experiences of homeschooling during the COVID-19 pandemic: differences between seven European countries and between children with and without mental health conditions. European Child & Adolescent Psychiatry. | Cross-sectional (Online survey) | Medium – some limitations |
|  |  | Gillespie-Smith, K. et al., 2021. The impact of COVID-19 restrictions on psychological distress in family caregivers of children with neurodevelopmental disability in the UK. | Cross-sectional paper | Strong |
|  |  | Toseeb, U.A., K; Code, A; Fox, L; Deniz, E, 2020. Supporting Families with Children with Special Educational Needs and Disabilities During COVID-19. | Cross-sectional (Online survey) | Medium – some limitations |
|  | Kinship carers and family caregivers | Grandparents Plus Charity, 2020. Kinship Care COVID-19 Impact Report. | Cross-sectional (Online survey) | Medium – some limitations |
|  |  | Family Rights Group: Deacon, 2020. Kinship Carers’ Experiences during the Coronavirus Crisis. | Cross-sectional (Online survey) | Medium – some limitations |
|  |  | Gallagher, S. and Wetherell, M.A., 2020. Risk of depression in family caregivers: unintended consequence of COVID-19. | Cross-sectional (Online survey) | Strong |
|  | Perinatal period | Davenport, M.H. et al., 2020. Moms Are Not OK: COVID-19 and Maternal Mental Health. | Cross-sectional (Online survey) | Medium – some limitations |
|  |  | Fallon, V. et al., 2021. Psychosocial experiences of postnatal women during the COVID-19 pandemic. A UK-wide study of prevalence rates and risk factors for clinically relevant depression and anxiety. | Cross-sectional (Online survey) | Medium – some limitations |
|  |  | Vazquez-Vazquez, A. et al., 2021. The impact of the Covid-19 lockdown on the experiences and feeding practices of new mothers in the UK: Preliminary data from the COVID-19 New Mum Study. | Pre-post pandemic between subjects design | Strong |
|  |  | Tomfohr-Madsen, L.M., Racine, N., Giesbrecht, G.F., Lebel, C. and Madigan, S. (2021). Depression and anxiety in pregnancy during COVID-19: A rapid review and meta-analysis. | Rapid review and meta-analysis | Strong |
| (2) Earning capacity changes | | | | |
| 7 | Economic harms | Save the Children, 2020. *Families in lockdown: Save The Children reports 56% of parents worried about their children’s mental health.* | Cross-sectional (Online survey) | Medium – some limitations |
|  |  | Adams-Prassl, A. et al., 2020. Furloughing*. | Cross-sectional (Online survey) | Strong |
|  |  | Dickerson, J. et al., 2021. Experiences of lockdown during the Covid-19 pandemic: descriptive findings from a survey of families in the Born in Bradford study | Cross-sectional (Online survey) | Strong |
|  | Kinship carers and family caregivers | Grandparents Plus Charity, 2020. Kinship Care COVID-19 Impact Report. | Cross-sectional (Survey) | Medium – some limitations |
|  |  | Family Rights Group: Deacon, 2020. Kinship Carers’ Experiences during the Coronavirus Crisis. | Cross-sectional (Online survey) | Medium – some limitations |
|  | Employment/  career opportunities | Parents and Carers in Performing Arts (PIPA), 2020. *COVID Report.* | Cross-sectional (Online survey) | Medium – some limitations |
|  |  | Myers, K.R., et al., 2020. Unequal effects of the COVID-19 pandemic on scientists. | Cross-sectional (Online survey) | Strong |
| (3) Physical harms – violence in the home | | | | |
| 4 | Domestic violence | Newbury, A. et al., 2020. Uned Atal Trais Violence Prevention Unit. *Understanding the Impact of COVID-19 on Violence and ACEs Experienced by Children and Young People in Wales.* | Literature review of multi-agency data | Medium – some limitations |
|  | Child and adolescent violence towards parents | Newbury, A. et al., 2020. Uned Atal Trais Violence Prevention Unit. *Understanding the Impact of COVID-19 on Violence and ACEs Experienced by Children and Young People in Wales.* | Literature review of multi-agency data | Medium – some limitations |
|  |  | Grandparents Plus Charity, 2020. Kinship Care COVID-19 Impact Report. | Cross-sectional (Survey) | Medium – some limitations |
|  |  | Condry, R. et al., 2020. Experiences of Child and Adolescent to Parent Violence in the COVID-19 Pandemic. | Cross-sectional (Online survey) and Freedom of Request summary from police forces. | Strong |

**More than 32 studies have been presented in the table due to some references being applicable to multiple harms.*

**Table S2.** Details of studies characteristics relating to harms

Presented in order of appearance in main text.

| Author, year | Location | Cohort | Sample size (N) | Age range of children | Data collection dates |
| --- | --- | --- | --- | --- | --- |
| 1. Mental health and well-being | | | | | |
| Psychological distress | | | | | |
| Pierce et al., 2020 | UK | Data from All household members aged 16 or older in April 2020, except for those unable to make an informed decision as a result of incapacity, and those with unknown postal addresses or addresses abroad. | 17,452 for COVID-19 web survey | N/A | April – May, 2020 |
| Xue & McMunn, 2021 | UK | Data from wave 9 (2017–19) of Understanding Society and the following April and May waves of Understanding Society Covid-19 study. All household members were aged 16 or older in April 2020. | 29,576 (April wave: 15,426, May wave: 14,150) | N/A | April – May, 2020 |
| Dickerson et al., 2021 | Bradford, UK | Survey data collected from the Born in Bradford studies with parents participating in two longitudinal studies: Bradford Growing Up (BiBGU) and Bradford’s Better Start (BiBBS) – although longitudinal data obtained before the pandemic have not yet been presented. | 2,144 | 0-13 years | 10th April - 30th June 2020 |
| Waite et al., 2020 | UK | Report 02 of the Co-SPACE longitudinal study (COVID-19: Supporting Parents, Adolescents and Children during Epidemics). Preliminary survey results from parents/carers of school-aged children. | 5,000 | 4-16 years | May, 2020 |
| Shum et al., 2020 | UK | Report 07 from the Co-SPACE longitudinal study (COVID-19: Supporting Parents, Adolescents and Children during Epidemics). Parents assessed at monthly intervals. Of the overall sample, 4,380 completed at least one follow-up, while 3,218 completed two or more follow ups. | 6,246 | 4-16 years | April - December, 2020 |
| McElroy et al., 2020 | UK | Convenience sample of parents and adolescents the Co-SPACE longitudinal study (COVID-19: Supporting Parents, Adolescents and Children during Epidemics). | 4,793 | 4–16 years | March - April 2020 |
| Well-being | | | | | |
| Royal Foundation of the Duke and Duchess of Cambridge, 2020 | UK | Parents and families of young children in the UK as part of a wider report to understand public attitudes towards the early years. Survey data collected in October 2020 (during the pandemic) was compared to data obtained September – February 2020 (before wide-spread restrictions). | 1,000 | 0-5 years | October, 2020 |
| El-Osta et al., 2021 | England, UK | Parents of school-age children in the UK (87% respondents were female). | 1,214 | 4 – 16 years | May – July, 2020 |
| Office for National Statistics, 2021 | UK | Experiences of adults with children that are being homeschooled because of school closures. Parents had at least one-school aged child (parents where all their children were aged between 0 and 4 were excluded. | 18,112 | 5 -18 years | February, 2021 |
| Bones et al., 2020 | Northern Ireland, UK | Parents of pupils attending primary (n=2,509), post-primary (n=1,905) and special schools (n=198). | 4,612 | 3-19 years old | April - May 2020 |
| Purdy et al., 2021 | Northern Ireland, UK | Parents/carers of children being home-schooled due to COVID-19 | 3,668 | <18 years | 9^th^ – 22^nd^ February, 2021 |
| Asbury et al., 2020 | UK | Parents or carers of school-aged children with Special Educational Needs and Disabilities (SEN/NDs) (92% were mothers; 95% from England with remainder from Scotland and Wales). | 241 | 5-18 years | March – April 2020 |
| Greenway & Eaton-Thomas, 2020 | UK | Parents home-schooling a child with special educational needs and disabilities (SEND) (95% were female with nearly half aged between 30-39 years). | 238 | 0-18 years | June – July 2020 |
| Toseeb et al., 2020 | UK | Parents of children with SENDs (the majority with Autism Spectrum Conditions). 91% were mothers and 96% were from England. | 239 | 5-18 years | March - May 2020 |
| Thorell et al., 2021 | UK, Sweden, Spain, Belgium, Netherlands, German, Italy | Parental experiences of home-schooling in families with or without a child with a mental health condition across seven European countries. | 6,720 (UK: 508) | 5–19 years | April - June 2020 |
| Parents of children with pre-existing medical conditions | | | | | |
| Darlington et al., 2020 | UK | Parents of a child with cancer (84% mothers). The majority of children were currently receiving treatment (67%). | 171 | 1-24 years | April 2020 |
| Parents of children with SEN/ND | | | | | |
| Waite et al., 2020 | UK | Initial results of survey data from parents/carers of school-aged children who are part of the Co-SPACE longitudinal study (COVID-19: Supporting Parents, Adolescents and Children during Epidemics). | 5000 | 4 – 16 years | May, 2020 |
| Shum et al., 2021 | UK | Report 09 from the Co-SPACE longitudinal study (COVID-19: Supporting Parents, Adolescents and Children during Epidemics). Parents assessed at monthly intervals. Of the overall sample, 4,557 have completed two or more follow ups (up to ten times so far). | 8,386 | 4-16 years | March 2020 - February 2021 |
| Gillespie-Smith et al., under review | UK | Caregivers of children with neurodevelopmental disabilities (n=43) and children who are typically developing (*n* = 67) | 110 | 11.2 years | April – June 2020 |
| Toseeb et al., 2020 | UK | Parents of children with SENDs (the majority with Autism Spectrum Conditions). 91% were mothers and 96% were from England. DID NOT compare two groups. | 239 | 5-18 years | March - May 2020 |
| Thorell et al., 2021 | UK, Sweden, Spain, Belgium, Netherlands, Germany, Italy | Parental experiences of home-schooling in families with or without a child with a mental health condition across seven European countries. | 6,720 (UK: 508) | 5–19 years | April - June 2020 |
| Kinship carers and family caregivers | | | | | |
| Grandparents Plus Charity, 2020. | England, UK | Kinship carers. | 169 | N/A | May 2020 |
| Family Rights Group, 2020 | Scotland, UK | Respondents who are raising kinship children and birth children. The majority (53%) were grandmothers. | 79 | 0 – 17 years | April 2020 |
| Gallagher & Wetherel, 2020 | UK | Data (1349 caregivers; 6178 non-caregivers) was extracted from Understanding Society, a UK population-level dataset. | 7527 | <18 years | May 2020 |
| Women during the perinatal period | | | | | |
| Davenport et al., 2020 | Worldwide: 8% of sample from UK | 900 eligible women: 520 (58%) were pregnant and 380 (42%) were in the first year after delivery. Mean age 33 years (range 17-49 years). | 900 | 0 – 5 years | April - May 2020 |
| Fallon et al., 2021 | UK | Sample of UK mothers. Mean age 31 years and 96% of white ethnicity. | 614 | 0–12 weeks | April – May 2020 |
| Vazquez-Vazquez et al., 2021 | UK | Women living in the UK aged ≥18 years (mean age 32 years; 94% were of white ethnicity and 95% were married/with partner). | 1,365 | <12 months | May - June 2020 |
| 1. Earning capacity changes | | | | | |
| Economic harms | | | | | |
| Save the Children Report | UK | UK parents with children aged 6-18 years. | 1,002 | 6-18 years | March 2020 |
| Adams-Prassl et al., 2020 | UK | UK residents at least 18 years old and reported having engaged in any paid work (including self-employment) during the previous 12 months. | 8940 (First wave: 4931, second wave: 4009) | N/A | Wave 1: April 2020  Wave 2: May 2020 |
| Dickerson et al., 2021 | Bradford, UK | Survey data collected from the Born in Bradford studies with parents participating in two longitudinal studies: Bradford Growing Up (BiBGU) and Bradford’s Better Start (BiBBS) – although longitudinal data obtained before the pandemic have not yet been presented. | 2,144 | 0 – 13 years | April - June 2020 |
| Kinship carers and family caregivers | | | | | |
| Grandparents Plus Charity, 2020. | England, UK | Kinship carers | 169 | N/A | May 2020 |
| Family Rights Group, 2020 | Scotland | Respondents who are raising kinship children and birth children. The majority (53%) were grandmothers. | 79 | 0 – 17 years | April 2020 |
| Employment/career opportunities | | | | | |
| Parents and carers in Performing Arts (PiPA), 2020 | UK | Performing arts workers who are parents or carers (91% parents and 14% cared for a disabled child or elderly, ill or disable adult). 80% of respondents were female. | 500 | N/A | September – October 2020. |
| Myers et al., 2021 | Worldwide | US and Europe based scientists across a wide range of institutions, career stages and demographic backgrounds. | 4,535 | N/A | April 2020 |
| 1. Physical harms – violence in the home | | | | | |
| Domestic violence | | | | | |
| Newbury, A. et al., 2020 | Wales, UK | Review of impact of COVID-19 on children and young people in Wales with a focus on violence and adverse childhood experiences. | N/A | N/A | Published Nov 2020 |
| Child and adolescent violence towards parents | | | | | |
| Newbury, A. et al., 2020 | Wales, UK | Review of impact of COVID-19 on children and young people in Wales with a focus on violence and adverse childhood experiences. | N/A | N/A | Published Nov 2020 |
| Grandparents Plus Charity, 2020. | England, UK | Kinship carers | 169 | N/A | May 2020 |
| Condry et al., 2020 | UK | Parents who have experiences of C/APV from their child. Also contains a summary of a Freedom of Information Request to all 43 police forces across England and Wales requesting numbers of reported C/APV incidents from April 2019 to May 2020. | 104 | 10 -19 years | April – June 2020 |

**More than 32 studies have been presented in the table due to some references being applicable to multiple harms.*
